# Clinical Characteristics of Coronavirus Disease 2019 (COVID-19) among Patients at a Movement Disorders Center

**DOI:** 10.3390/geriatrics5030054

**Published:** 2020-09-18

**Authors:** Joy Antonelle de Marcaida, Jeffrey Lahrmann, Duarte Machado, Lawrence Bluth, Michelle Dagostine, Maria Moro-de Casillas, Elena Bortan, Sulada Kanchana, Mark Alberts

**Affiliations:** 1 Chase Family Movement Disorders Center, Hartford HealthCare, Hartford, CT 06066, USA; Jeffrey.Lahrmann@hhchealth.org (J.L.); Duarte.Machado@hhchealth.org (D.M.); Michelle.Dagostine@hhchealth.org (M.D.); Maria.Moro-de-Casillas@hhchealth.org (M.M.-d.C.); Elena.Bortan@hhchealth.org (E.B.); Sulada.Kanchana@hhchealth.org (S.K.); 2 Hartford Neurology LLC, Hartford, CT 06066, USA; Lawrence.Bluth@hhchealth.org; 3 Ayer Neuroscience Institute, Hartford HealthCare, Hartford, CT 06066, USA; Mark.Alberts@hhchealth.org

**Keywords:** coronavirus, COVID-19, neurology, movement disorders, ataxia, adamantanes, Parkinson disease

## Abstract

It is not established whether SARS-CoV-2 (COVID-19) patients with movement disorders, are at greater risk for more serious outcomes than the larger COVID-19 population beyond the susceptibility associated with greater age. We reviewed electronic health records and conducted telephone interviews to collect the demographics and clinical outcomes of patients seen at our Movement Disorders Center who tested positive for COVID-19 from 8 March 2020 through 6 June 2020. Thirty-six patients were identified, 23 men and 13 women, median age of 74.5 years. They primarily carried diagnoses of idiopathic Parkinson disease (*n* = 22; 61%) and atypical parkinsonism (*n* = 7; 19%) with the balance having other diagnoses. Twenty-seven patients (75%) exhibited alteration in mental status and fifteen (42%) had abnormalities of movement as common manifestations of COVID-19; in 61% and 31%, respectively, these were the presenting symptoms of the disease. Sixty-seven percent of patients in our cohort required hospitalization, and the mortality rate was 36%. These data demonstrate that in patients with movement disorders, the likelihood of hospitalization and death after contracting COVID-19 was greater than in the general population. Patients with movement disorders frequently presented with altered mental status, generalized weakness, or worsening mobility but not anosmia.

## 1. Introduction

Coronavirus disease 2019 (COVID-19) is a novel respiratory illness first reported in December 2019 [1]. The manifestations of COVID-19 may range from mild (or asymptomatic) to severe illness leading to respiratory failure and death [2]. The current literature suggests that the factors associated with worse outcomes include older age (>50 years), male sex, living in long-term care facilities, and medical comorbidities such as cardiovascular disease, hypertension, diabetes, chronic lung disease, renal disease, and immunosuppression [3,4]. Parkinson disease (PD) and other movement disorders have not been reported as particular risk factors for more serious sequelae from COVID-19 to date. However, because the patients cared for at specialized Movement Disorders programs are frequently older, tend to have an increased incidence of physical comorbidities (including identified risk factors for more severe manifestations of COVID-19), have increased frailty, and are more likely to be residents of long-term care facilities [5,6,7,8,9], concerns were raised early in the pandemic that this patient population may be particularly vulnerable to the disease [5,6,10,11].

The first patient with laboratory-confirmed COVID-19 in the State of Connecticut was diagnosed on 8 March 2020, and by 6 June 2020, the time of this reporting, there were 4.3818 × 10^4^ cases in the state [12]. Thus, as the number of cases infected with severe acute respiratory syndrome coronavirus 2 (SARS-CoV-2) increased in our region, our team at Hartford HealthCare’s Chase Family Movement Disorders Center implemented immediate initiatives, consistent with the early recommendations published in Movement Disorders journals, to mitigate the risk to our patients [5,7,13], such as early conversion to telemedicine visits, conducting several bilingual virtual educational COVID-19 lectures, providing opportunities to participate in virtual exercise classes and support groups, supplying free face masks to patients and their caregivers if needed, and monitoring for symptoms of COVID-19 infection. Despite these measures, we identified that thirty-six patients of our program tested positive for COVID-19 from 8 March 2020 to 6 June 2020. In this report, we describe the demographic characteristics, presentation, management, and outcome of these patients, with the intent of exploring factors that may influence the clinical course in this patient population.

## 2. Materials and Methods

The cohort consists of two overlapping groups of patients. First, we are including patients of the Chase Family Movement Disorders Center (CFMDC), an ambulatory program affiliated with Hartford HealthCare (HHC), specializing in the care of people with Parkinson disease and other movement disorders. The Center has three locations throughout Connecticut, attending to over 3000 patients with PD and parkinsonism, and over 8900 total patients with all movement disorders. We gathered information on our patients who tested positive for COVID-19 from 8 March 2020 through 6 June 2020, spanning ninety days from the time that the first COVID-19 case was reported in Connecticut. The Center was alerted of patients who contracted COVID-19 by family members, caregivers, extended care facility staff, and physicians at other practices and regional hospitals, or when our own staff and clinicians called the patient for their follow-up telemedicine visit. We did not do a complete survey of our entire patient population. We then conducted telephone interviews and reviewed medical charts from the hospital electronic health record systems (EHRs) to obtain all pertinent data for this paper. Second, we reviewed Hartford HealthCare’s EHR to identify patients with Parkinson disease who were admitted for COVID-19 to any of our six affiliate hospitals across the state as a way to estimate the burden of COVID-19 on the population of patients with movement disorders. In each of these cases, the diagnosis of COVID-19 was established using reverse transcriptase polymerase chain reaction (rt-PCR) tests that detected nucleic acid from SARS-CoV-2 from respiratory specimen. By including patients who were identified through routine telemedicine visits, self-reporting, and inpatient registry review of the HHC EHR, we captured information on a broad spectrum of the population and minimized selection bias that can occur when conducting an exclusively community-based or inpatient hospital-based review.

This retrospective study has been approved by Hartford HealthCare’s IRB (HHC-2020-0179). All relevant de-identified data will be available to qualified investigators upon request.

## 3. Results

Of the thirty-six cases identified with COVID-19 (Table 1), twenty-three (64%) were men and thirteen (36%) were women. The median age was 74.5 (range 33 to 90 years old), with majority (89%) being over 60 years old. The patients in this cohort carried the following diagnoses (*n*; %): idiopathic Parkinson disease without dementia (*n* = 8; 22%), idiopathic Parkinson disease with dementia (*n* = 14, 39%), atypical parkinsonism (*n* = 7; 19%), vascular parkinsonism (*n* = 1; 3%), Tourette syndrome (*n* = 2; 5%), neurodegeneration with brain iron accumulation type 5 (*n* = 1; 3%), multifactorial gait disorder (*n* = 1; 3%), essential tremor (*n* = 1; 3%), and post-COVID-19 cerebellar ataxia (*n* = 1; 3%).

This last patient (patient 21) did not have a prior diagnosis of a movement disorder, but rather was referred to our program for new-onset cerebellar ataxia. He presented one month prior to our evaluation, with fever, cough, shortness of breath, and fatigue, but was not tested for COVID-19 at that time. His wife developed similar symptoms two days later, and she tested positive for SARS-CoV-2. He recovered after 11 days and returned to work. About a week later, he developed symptoms of cerebellar dysfunction, including disabling tremors, gait instability, dysarthria, and vertigo. He also complained of mild short-term memory impairment. On presentation to our center, he had notable gait and left upper extremity ataxia, but vertigo had resolved, and tremors were mild. He tested positive on both the rt-PCR and antibody testing for SARS-CoV-2. Electrophysiologic testing was negative for evidence of an acute inflammatory demyelinating polyradiculopathy. He declined a spinal tap. Possible autoimmune, paraneoplastic and metabolic causes of subacute ataxia were negative on laboratory testing. Cranial MRI showed mild prominence of cerebellar vasculature at the surface, which was described as nonspecific, but possibly seen with an inflammatory etiology. A diagnosis of post-viral cerebellitis related to COVID-19 was proposed. He continued to improve, without any further intervention, over the next few weeks. By the time of this reporting, most symptoms had almost completely resolved.

Twenty-eight patients (78%) had at least one of six comorbidities associated with more severe COVID-19 (hypertension, cardiovascular disease, renal disease, diabetes, chronic lung disease, or immunosuppression), with the majority having more than one risk factor. Thirteen patients (36%) lived in their own home, while twenty-three (64%) were at extended care facilities: nursing home (*n* = 17), assisted living facility (*n* = 5), and group home (*n* = 1). Their presentation, clinical course, management, and outcome are also presented in Table 1. Of interest, twenty-seven patients (75%) exhibited alteration in mental status (including lethargy, confusion, delirium, hallucinations, or bradyphrenia), and in twenty-two (61%), it was the presenting symptom. Of these, only fifteen (56%) had a pre-existing diagnosis of dementia. Thirteen patients (36%) were documented to have hypotension, and fifteen (42%) were noted to have abnormalities of movement. Worsening of their movement disorder was reported by seven patients (19%), and generalized weakness or worsening mobility were noted as the presenting symptom by eleven patients (31%). Twenty-four patients (67%) required hospitalization. Nine patients received hydroxychloroquine and two received oseltamivir. Six patients were receiving an adamantane as part of their neurologic management (three on amantadine, three on memantine).

**Figure 1:**
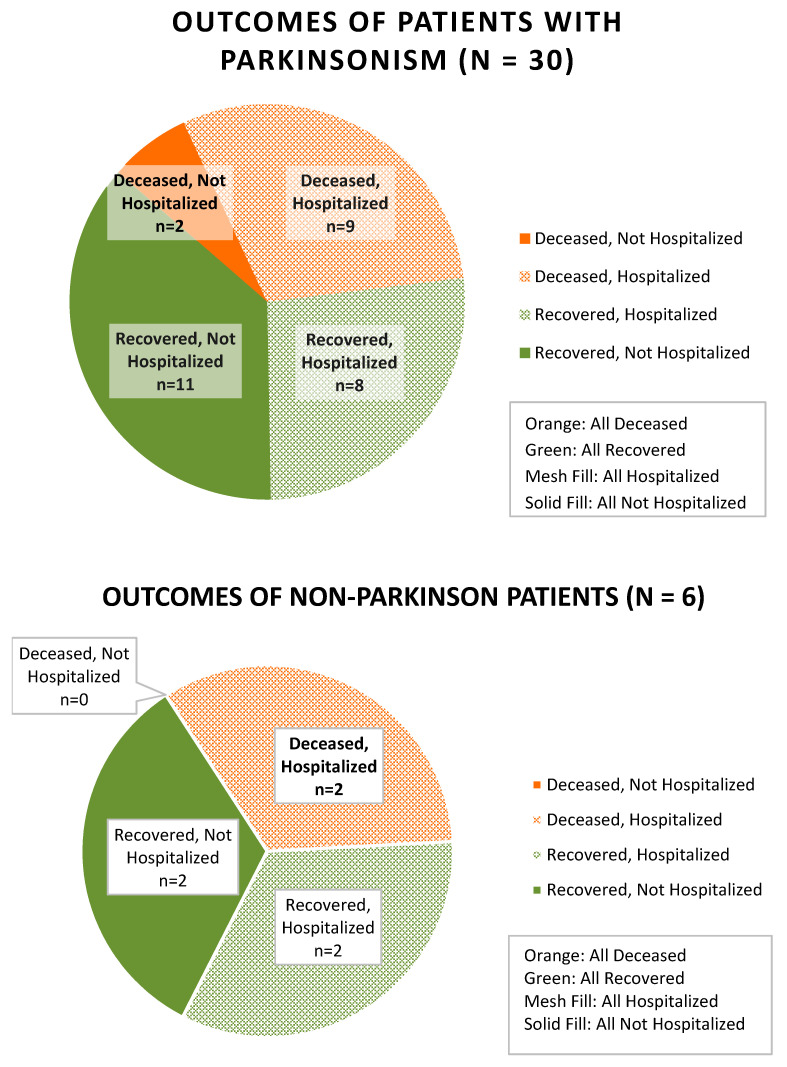
Outcomes of patients with parkinsonism versus without parkinsonism.

Mortality in this series was thirteen out of thirty-six patients (36%). Of the patients who died, twelve (92%) were greater than 60 years old, eleven (85%) had parkinsonism, eleven (85%) were from an extended care facility, nine (69%) had comorbid dementia, nine (69%) had at least one high-risk comorbid condition, eight (62%) had alteration in mental status as a presenting symptom, and eight (62%) received a medication with antiviral properties (hydroxychloroquine, oseltamivir, amantadine, and memantine), including three who were on an adamantane prior to contracting COVID-19. Looking specifically at the patients in our cohort with parkinsonism, versus those without parkinsonism, their outcomes are separated out and shown in Figure 1.

Lastly, upon review of the inpatient database of Hartford HealthCare’s six affiliate hospitals throughout the state, we found that of 2028 patients admitted for COVID-19 within the same 90-day period of our study, forty-two patients (2%) carried a diagnosis of PD or a parkinsonism. Only sixteen of these forty-two patients (38%) admitted to the HHC hospital network were patients of the Chase Family Movement Disorders Center.

## 4. Discussion

The increased susceptibility to COVID-19 in patients with Parkinson disease and other movement disorders has been presumed and was a source of heightened concern in the early months of this pandemic [5,6,10]. The inherent characteristics and comorbidities of this population made it quite plausible that these patients are particularly vulnerable. Older age, preponderance in males, increased likelihood of comorbid cardiovascular diseases, and increased risk of respiratory dysfunction had all been cited as potential risk factors for worse outcomes in this patient population, particularly patients with Parkinson disease, from COVID-19. Furthermore, it has been reported that patients with PD may have cognitive and motor inflexibility, impaired activation of stress response mechanisms, and a susceptibility to adverse effects of social isolation and loss of physical activity that render them less likely to cope well with the consequences of this pandemic [5,6,9]. Up until recently, however, systematic data did not show an apparent increased risk of contracting COVID-19 or for worse outcomes of the disease in people with PD [5,6]. A community-based observational study of 141 patients with Parkinson disease in Lombardy, one of the most heavily affected regions in Italy by COVID-19, yielded 12 cases or an 8.5% incidence of infection [14]. The authors noted that there were no deaths from COVID-19 in their cohort, and patients manifested primarily with mild to moderate symptoms not requiring hospitalization except for one patient. On the other hand, Antonini and colleagues reported on the outcomes of 10 PD patients affected by COVID-19 at the Parkinson and Movement Disorders unit in Italy and at King’s College Hospital in London, and suggested that PD patients, particularly those who are older and on advanced therapies, should be considered as a specifically susceptible group because of the high mortality rate noted for this subset of patients in their series [15].

Our study provides information on a cohort that is three times larger than that of the previously cited articles, collected within a similar span of time. Our cohort, however, includes a wide variety of patients with movement disorders and not exclusively patients with a diagnosis of PD. Regardless, in this series, we found that within a Movement Disorders subspecialty practice, 81% of the patients who developed COVID-19, and 85% of the patients who died, were people with PD or a parkinsonism. Whether this is because of an actual increased risk of COVID-19 in patients with Parkinson disease, or whether it is because Movement Disorders programs tend to see more people with PD than other movement disorders, is difficult to ascertain. At the CFMDC, people with PD and parkinsonism comprise one third of all the patients we see at all three of our locations, which is disproportionate to the number of patients with parkinsonism who developed COVID-19 in this cohort.

Similar to the observations in patients who developed COVID-19 in the general population, our cohort was predominantly male (64%), 60 years of age or older (89%), had high-risk comorbidities for COVID-19 (78%), and most resided at an extended care facility (64%). Of note, twenty-one (58%) of the patients in this cohort also had comorbid dementia. A recent retrospective study of 627 subjects admitted to an acute hospital in northern Italy yielded 82 patients (13%) who carried a prior diagnosis of dementia, but with a staggering 62% mortality rate, as compared to 26% in patients without dementia at the same institution [16]. Of the thirteen patients in our cohort who died, nine (69%) had comorbid dementia. The overall mortality rate in our cohort for patients with comorbid dementia was 43%. This was higher than the mortality rate of patients with a parkinsonism in this cohort, which was 37%. In comparison, a study of 191 inpatients with COVID-19 in Wuhan, China, yielded a mortality rate of 67% for those with known high-risk medical comorbidities, and 33% for those without any of these comorbidities [3]. In two large studies, overall mortality rates for patients with COVID-19 admitted to hospitals in China and the United Kingdom were 28% and 26%, respectively [3,17]. Thus, although dementia and parkinsonism in our cohort were associated with significant mortality, higher than that reported in general populations, the rates were not as high as has been reported in patients with other comorbidities known to correlate with unfavorable outcomes. Within our cohort of patients with movement disorders, factors related to increased mortality were age over 60 years, PD or parkinsonism diagnosis, residing in an extended care facility, comorbid dementia, and comorbid medical conditions.

As regards the clinical manifestation and disease course of patients in this cohort, we found that two-thirds of patients (67%) required hospitalization, almost three times higher than the reported 23% hospitalization rate among the general population in Connecticut [18]. This observation supports the hypothesis that patients with movement disorders are at particular risk for adverse outcomes with COVID-19. In terms of specific clinical presentation, we found that alteration in mental status, generalized weakness, worsening mobility or the motor symptoms of the underlying movement disorder, and hypotension were common manifestations of COVID-19. In this series, fever, cough, dyspnea, and malaise were almost universally present, but 61% presented with alteration in mental status, and 31% with worsening mobility and/or balance as one of the primary symptoms leading to SARS-CoV-2 testing. Encephalopathy has increasingly been recognized as a presenting symptom of COVID-19, and gait instability is a common presentation of hospitalized patients with movement disorders who develop acute infectious-metabolic conditions [19,20,21]. Anosmia was reported by only one patient who did not have a pre-existing diagnosis of a movement disorder but was referred to our program for new-onset cerebellar ataxia. We postulate that patients with a pre-existing parkinsonism or dementia are less likely to report anosmia as a presenting symptom of COVID-19 because many already have long-standing anosmia related to their neurodegenerative condition.

Three of the patients in this series were on amantadine as part of the management of their parkinsonism, and three were on memantine for dementia. There has been an interest in the potential of adamantanes to alter the course of COVID-19 based on their ability to interfere with viroporin protein channels responsible for the release of RNA-viruses from infected cells [22]. Amantadine in particular was also recently discovered to downregulate the expression CTSL gene coding for the cathepsin L, a lysosomal protease involved in SARS-CoV-2 entry into cells [23]. Based on these potential antiviral effects, it was proposed that adamantanes could serve as a potent therapeutic, decreasing the replication and infectivity of the virus, and possibly leading to better clinical outcomes. Rejdak and colleagues then identified 22 patients who tested positive for SARS-CoV-2 and were taking either amantadine or memantine, and reported that none of these patients developed any clinical manifestations of COVID-19, nor did they report any significant change in their neurologic status [24]. In our cohort, we had six patients who were taking adamantanes who developed significant symptoms related to COVID-19, and three of them died.

In summary, our study supports the recommendation that clinicians must remain vigilant for potential acute and chronic complications of COVID-19 when caring for patients with Parkinson disease and other movement disorders because of the inherent vulnerabilities of this patient population. Our cohort yielded several interesting observations that either confirmed or negated previous assumptions and findings reported regarding COVID-19 in patients with PD and other movement disorders. Within a large database of inpatient cases throughout our hospital system, people with PD and parkinsonism accounted for only 2% of patients admitted for COVID-19. However, for patients with movement disorders, the likelihood of hospitalization after contracting COVID-19 was three times higher than that of the general population. Furthermore, we found that older age, PD diagnosis, living in an extended care facility, comorbid dementia, and comorbid medical conditions were associated with more serious morbidity and death. Even so, the mortality rate for patients with PD or dementia in this cohort was significantly less than the mortality previously reported in patients with other high-risk comorbidities, although higher than that reported in general populations. We also noted that patients with movement disorders frequently presented with altered mental status, generalized weakness, or worsening mobility, but not anosmia, as the initial symptoms of COVID-19. Lastly, within this limited dataset, we did not observe that amantadine or memantine afforded distinct protective properties against COVID-19, as was suggested in the paper by Redjak et al. [24].

## 5. Conclusions

We recognize that the major limitation of our study is the retrospective data collection from a comprehensive (hospital) data source and self-reports from our Movement Disorders Center patients. Furthermore, although our cohort is larger than others previously reported, the sample size of 36 patients still precluded us from performing multivariate adjusted analyses. Despite these limitations, our findings contribute further to understanding the clinical course of COVID-19 among patients with movement disorders. Future multicenter studies will be needed to create a large enough dataset to carry out rigorous statistical analyses to determine the specific vulnerabilities of patients with movement disorders.

## Figures and Tables

**Table 1:** Patient Characteristics, Clinical Course, and Outcomes.

Patient	Age/Sex	Living Situation	Movement Disorder (Duration in Years)	Comorbidities	Clinical Picture Requiring SARS-CoV-2 Testing	Signs/Symptoms Developed during Clinical Course	Hospital Admission (Yes/No)	Pharmacological Interventions	Outcome
**1**	79/M	House	Parkinson disease with Dementia (13 years)	HTN, CKD stage 3, prostate cancer	fever, rigors, emesis, diarrhea, lethargy, confusion, ARDS, hypotension	lethargy confusion hypotension	Yes	Amantadine 100 mg BID *	Died
**2**	72/M	ECF (NH)	Hydrocephalus, Secondary Spasticity (DDD, Stroke), multifactorial gait disorder (3 years)	HTN, strokes	nasal congestion, lethargy, generalized weakness, fever	lethargy confusion hypotension	Yes	Hydroxychloroquine, Memantine 10 mg BID *	Died
**3**	83/M	ECF (NH)	Parkinson disease with Dementia (8 years)	HTN, CAD, DM2	dyspnea, fever, lethargy, delirium, confusion, ARDS	lethargy, delirium confusion, hallucinations, myoclonus, asterixis, hypotension	Yes	Oseltamivir	Died
**4**	67/M	Apartment	Parkinson disease (10 years)	HTN, CAD, DM2, cardiomyopathy	dyspnea, diarrhea, lethargy, nausea, vomiting	lethargy, worsening of gait instability, worsening of tremors	No	Amantadine 100 mg BID *	Recovered
**5**	40/M	ECF (NH)	Parkinsonism, Ataxia (28 years)	HTN	could not obtain	could not obtain	No	Amantadine 137 mg qHS *	Recovered
**6**	71/F	House	Parkinson disease (3 years)	DM2, meningioma	generalized weakness, diarrhea, dizziness, lethargy	lethargy, delirium, bradyphrenia, dizziness, generalized weakness	Yes		Recovered
**7**	77/F	ECF (NH)	Parkinson disease with Dementia and Psychosis (8 years)	Afib, nOH	lethargy, cough, dyspnea	Lethargy	No		Recovered
**8**	77/M	House	Parkinson disease (3 years)	nOH, CKD stage 3, LBBB CVD	lethargy, cough, dyspnea, loss of appetite, fever	lethargy, hypotension	Yes		Recovered
**9**	33/F	ECF (GH)	Neurodegeneration with Brain Iron Accumulation type 5 with Dementia (15 years)		dyspnea, cough, fever	lethargy, hypotension	Yes	Hydroxychloroquine	Died
**10**	82/F	ECF (NH)	Parkinson disease (11 years)	SDH, bladder cancer, prior ETOH abuse	gait instability with falls, lethargy, fever, hypotension	lethargy, confusion, worsening of gait instability, hypotension	Yes		Recovered
**11**	69/M	ECF (NH)	Parkinson disease with Dementia (6 years)	Strokes, CAD, HTN	dyspnea, fever, lethargy, confusion, ARDS	lethargy, confusion, hypotension	Yes	Hydroxychloroquine	Died
**12**	68/M	ECF (NH)	Parkinson disease (10 years)	PVD	fever, cough, dyspnea		No	Hydroxychloroquine	Died
**13**	77/M	House	Parkinson disease with Dementia (6 years)	HTN, CKD stage 3, Atrioventricular Block	Asymptomatic	lethargy, delirium, confusion, worsening of gait instability, syncope, generalized weakness	Yes		Recovered
**14**	77/M	ECF (ALF)	Lewy Body Dementia (9 years)	HTN, CAD, RBBB CVD, paroxysmal Afib, DM2	fever, confusion, lethargy, hypoxia, hypotension	lethargy, confusion, poor concentration, hypotension	Yes	Memantine 10 mg BID *	Recovered
**15**	87/M	House	Lewy Body Dementia (3 years)	HTN, PAD, Basal cell carcinoma	cough, body aches	delirium, urinary incontinence	No		Recovered
**16**	61/M	ECF (ALF)	Lewy Body Dementia (19 years)	nOH	lethargy, confusion, delirium, generalized weakness, hypotension, cough, dyspnea	lethargy, confusion, delirium, hypotension	Yes		Died
**17**	76/M	ECF (NH)	Parkinson disease with Dementia and Psychosis (8 years)	HTN, CAD, LBBB CVD, CKD	cough, dyspnea, lethargy, confusion, worsening of tremors	lethargy, confusion, worsening of gait instability, worsening of tremors, hypotension	Yes		Recovered
**18**	68/F	ECF (NH)	Progressive Supranuclear Palsy (5 years)	DM2, Breast Cancer, Renal Cell Carcinoma	could not obtain	could not obtain	No	could not obtain	Died
**19**	55/F	House	Tourette Syndrome (45 years)		generalized weakness, dyspnea, cough	generalized weakness	Yes		Recovered
**20**	72/M	House	Progressive Supranuclear Palsy with Dementia (5 years)	Cervical Dystonia	fever	could not obtain	No	could not obtain	Died
**21**	59/M	House	Cerebellitis due to SARS-CoV-2 Infection (0 years)	HTN	anosmia, dyspnea, cough, fever, lethargy	lethargy, confusion, bradyphrenia, ataxia, gait instability, anosmia	No		Recovered
**22**	90/M	ECF (NH)	Lewy Body Dementia (3.5 years)	HTN, Afib, CAD, Skin Cancer	cough, nasal congestion		No		Recovered
**23**	70/F	House	Essential Tremor (40 years)	Mantle Cell Lymphoma (on ibrutinib, s/p stem cell transplant), HTN, DM2	generalized weakness, lethargy, fever, cough, dyspnea, anorexia	lethargy, generalized weakness, hypotension, worsening of tremors	Yes	Hydroxychloroquine	Recovered
**24**	81/F	ECF (ALF)	Parkinson disease with Dementia (5 years)	HTN, Rheumatoid Arthritis (on Methotrexate)	dyspnea, cough	confusion	Yes		Recovered
**25**	74/M	ECF (NH)	Parkinson disease with Dementia (19 years)		could not obtain	could not obtain	No	could not obtain	Recovered
**26**	65/F	House	Tourette Syndrome (52 years)	Sarcoidosis, Asthma, Afib	cough, myalgia, lethargy, fever	lethargy	No	Oseltamivir	Recovered
**27**	87/M	ECF (ALF)	Vascular Parkinsonism (23 years)	Afib, COPD, CHF, PVD, DM2, HTN, CKD Stage 3, history of melanoma, history of bladder cancer	fever, cough, dyspnea, myalgia, generalized weakness, lethargy	confusion, lethargy, bradyphrenia, generalized weakness	Yes		Died
**28**	65/F	ECF (NH)	Parkinson disease (10 years)	HTN, CHF, prior PE, PVD, history of ovarian cancer	lethargy, generalized weakness, myalgia, fever	generalized weakness, lethargy	Yes		Recovered
**29**	75/F	ECF (NH)	Parkinson disease with Dementia and Psychosis (12 years)		Fever, dyspnea, myalgia, generalized weakness, confusion	confusion, generalized weakness, hypotension	Yes		Died
**30**	82/M	House	Parkinson disease (5 years)	Afib, Atrioventricular Block, CKD stage 4	cough, fever, anorexia, confusion	confusion	Yes	Hydroxychloroquine	Recovered
**31**	69/M	ECF (NH)	Parkinson disease with Dementia, Tardive Dyskinesia (23 years)		delirium, lethargy, dyspnea, diarrhea	delirium, lethargy	No		Recovered
**32**	80/F	ECF (NH)	Parkinson disease with Dementia, Normal Pressure Hydrocephalus (10 years)	CAD, CHF, DM2, HTN	cough, lethargy, confusion, dyspnea, hypoxia, abdominal pain	lethargy, confusion	Yes		Recovered
**33**	78/F	ECF (NH)	Parkinson disease with Dementia (28 years)	nOH, HTN	dyspnea, hypoxia	confusion	Yes		Died
**34**	71/M	House	Parkinson disease with Dementia (3 years)	ESLD, HTN, DM2, CHF, CKD Stage 5	dyspnea, cough, generalized weakness, confusion, delirium, lethargy	confusion, delirium, generalized weakness, lethargy	Yes	Hydroxychloroquine	Recovered
**35**	78/M	ECF (NH)	Parkinson disease with Dementia (8 years)	CAD, paroxysmal Afib, cardiomyopathy, HTN, ischemic stroke	fever, dyspnea, cough, hypoxia	hypotension	Yes	Hydroxychloroquine	Recovered
**36**	83/M	ECF (ALF)	Lewy Body Dementia (10 years)	COPD	fever, dyspnea, hypoxia		Yes	Hydroxychloroquine, Memantine 28 mg BID *	Died

Abbreviations: Afib: atrial fibrillation; ALF: assisted living facility; ARDS: acute respiratory distress syndrome; CAD: coronary artery disease; CKD: chronic kidney disease; CHF: congestive heart failure; COPD: chronic obstructive pulmonary disease; CVD: cardiovascular disease; DDD: degenerative disc disease; DM2: diabetes mellitus type 2; ECF: extended care facility; ESLD: end-stage liver disease; ETOH: alcohol; GH: group home; HTN: hypertension; NH: nursing home; nOH: neurogenic orthostatic hypotension; PAD: peripheral arterial disease; PVD: peripheral vascular disease; PE: pulmonary embolism; SDH: subdural hematoma. * patient receiving amantadine or memantine prior to hospitalization as part of treatment for movement disorder.
